# American Dental Association

**Published:** 1883-09

**Authors:** J. A. Robinson


### American Dental Association.

BY J. A. ROBINSON, D.D.S.

The twenty-first annual meeting is over and gone as far as the gathering together of men is concerned; but that is only an incident in comparison with the progress that has been made in the elevation and consolidation of thought in our profession. If we can measure the intensity, and the breadth of thought by location, then the West far outstrips the East in interest. There was but one representative from Boston, and only two from Massachusetts; while from New Jersey, Pennsylvania, New York, Ohio, Illinois, Michigan, Iowa, Texas, Louisiana, and still further west, sent a much larger number of delegates, in proportion to the number engaged in the profession. The educational interests were well cared for in the meeting of the Examining Boards, that met at the same time from these States where there are laws to protect the people from charlatantry and ignorance of unscrupulous practitioners in dentistry, which is a *lift* out of some of the difficulties of the profession, and a protection against the wholesale "slaughter of the innocents" that has been practiced by unscrupulous persons who are unfit to pursue one of the most useful and ornamental of all the professions.

The State has a right to demand service of the people, and should see to it that they are fit to serve; and no person can be fit to perform laborious service without good teeth.

The plan of dividing the work into sections is a good one, but the difficulty of placing the right men upon committees and sections can hardly be estimated. Every man will fill the pattern which his soul sees; and he had rather be like the philosopher at Athens, and drink the hemlock than vary one jot or title from his plan or purpose. So through the lack of missionary spirit that belongs to really great men, the Association is deprived of some of the good things that are brought to the meeting.

Every man is interested in his specialty, and it takes a large mind to grasp the whole subject. This was plainly seen by the comparatively few listeners to Dr. Harlan's address on the relative value of remedies in the treatment of Pyorrhœa Alveolaris, and more marked still, during the reading of the papers on Dental Literature and Nomenclature which bore the marks of scholarship, labor, and thought; and Dr. Fredrick's, though a good deal shorter, was concise and clear. Atkinson's paper led the van as he always does in hope and prophetic vision. The imperfection of our experiences suggests to him the absolute perfection, he is willing to pass over the forty years in pursuit of the promised land; and Dr. Harlan, when questioned about re-producing lost tissue, was not quite willing to stand up like Dr. Atkinson; but rather say the tissue was restored than re-produced.

It has always been true that our greatest men are our most willing learners. There is another class of self-satisfied men in every profession, men who think they have entered and possessed the Promised Land, and look with contempt on every person who proposes any innovation or improvement, and think the time wasted that is spent in Literature or Nomenclature. Dr. Crouse is a good representative of this class of men. He is not to blame for this, and his speeches are an index of his calibre in every direction. His offiensive tirade against the Dental Journals is conclusive evidence that he does not read or understand. The fact that he was absent from the meeting during most of the reading of the papers on Dental Literature and Nomenclature, and in a scurrilous speech wanted the papers of the section passed without knowing what they contained, was an offence that ought not to be overlooked. The brakes on the engine may sometimes ensure safety, but they are very insignificant compared to the grand locomotive that moves the world. There is an ancient theory that men are like what they feed upon. If this is true what do some persons eat? We do not find fault with Crouse so much for his lack of taste, and his dislike of some things, as because he is unwilling to listen to the minute facts in dental science, that those who are willing to bring before the association have found, and that seem to be above his appreciation. The road toward education is beneficent to the student. It is a process of growing towards the future, and is never completed as to tense. Nothing more sad can overtake a man than to see nothing before him in the future but so many dollars. If we all felt so, our contest in science would be ended here, and there would be no eternity to beckon us on.

The paper of Dr. Bödecker, of New York, was listened to with the closest attention; and his modest answers to the questions on the action of arsenious acid upon dentinal and pulp tissue were those of a man, who had measured every step he had taken in his investigation, with the microscope, and what he did not find he answered with "I did not see, and I do not know," which is the true measure of a strictly scientific man. Dr. Barrett, of Buffalo, made an extemporaneous report on Physiology and Etiology, and his remarks were forcible and clear as they are always. Dr. Elwin Richardson, of London, Eng., was introduced to the meeting; he said he came to hear a good deal, and to say very little—he came to learn and not to talk.

The steps in the science of dentistry that have been trod with so much care by the fathers in our profession are still nothing but the bridle paths that will lead to the great highway; and those who are unwilling to follow will be left behind, in ignorance and disappointment. The remarks of Prof. Kingsley, of New York, and his illustrations on the blackboard of the obturator and the art of speech, with the appliances, showed that labor and toil are not without compensation, and that a lifetime devoted to details in any particular branch of our profession is sure of a rich reward. Kingsley's dreams have become realities, and are, as the rich tones of his voice, a music to every one who will listen and hear. The Clinics were well attended, and were highly gratifying to all who are interested in good fillings or supplying artificial crowns upon the roots of teeth. Each operator had his specialty, and the skill and manipulation, and the excellent characteristics of the Bonwill engine and mallet; the Brown clamp and the Hodge hand piece, were all steps that mark the great progress that has been made in dental science and mechanics in the past few years. We speak particularly of these things because they seem to be outside of the Dental Depots. The display of dental goods by the various dental dealers was very good indeed. The election of officers was admirably conducted and good men fill the offices as a rule. The past history of our profession is fragmentary. It is not far removed from the hieroglyphs and rude alphabet that is the beginning of all science; but the fragments, brought together and consolidated by the brave workers, will place us among the foremost deliverers from the imperfections of humanity, as naturally as the fruits of autumn are filled with the sunshine, and the rain, and storms of the summer days.

